# A Case Report on VT from TV: DVT and PE from Prolonged Television Watching

**DOI:** 10.1155/2017/9347693

**Published:** 2017-10-03

**Authors:** Alan Lucerna, James Espinosa, Lindsey Ackley, Philip Carhart, Douglas Stranges, Risha Hertz

**Affiliations:** ^1^Emergency Department, Rowan University SOM, Kennedy University Hospital, Stratford, NJ 08084, USA; ^2^Emergency Department, Kennedy University Hospital, Stratford, NJ 08084, USA; ^3^University of Pennsylvania Health System, Penn Medicine, Gibbsboro, NJ, USA

## Abstract

Pulmonary embolus (PE) and deep vein thrombosis are diagnoses that are commonly made in the emergency department. Well known risk factors for thromboembolic events include immobility, malignancy, pregnancy, surgery, and acquired or inherited thrombophilias, obesity, cigarette smoking, and hypertension. We present a case of a 59-year-old female who watched TV and developed leg swelling and was found to have PE and DVT.

## 1. Case Report

A 59-year-old female with a past medical history of obesity (body mass index 51 kg/m^2^), autoimmune hepatitis, and osteoporosis presented to the emergency department (ED) complaining of right calf pain and swelling. She had no history of previous venous thromboembolic disease. The patient stated that the pain started after watching television for eight continuous hours. The following day, she noted persistent right calf pain. However, she was particularly engaged in national convention coverage and watched television continuously for approximately eight more hours. She recalls that she did not take any breaks from watching the convention; in fact she states she only moved from the chair once to go to the bathroom. After two days of mild throbbing pain and swelling, she decided to have family members drive her to the ED. After obtaining a history from the patient, she disclosed that she was having occasional dizziness and dyspnea on exertion.

The patient has a history of immune hepatitis which had been stable for years, treated with Imuran. Her surveillance liver function tests have been within normal limits for a while and she did not need adjustments of her medications. Her other medical history included hiatal hernia, sleep apnea requiring nighttime continuous positive airway pressure (CPAP) machine, and osteoporosis. She had no previous surgeries other than a left wrist surgery 20 years previously. She never smoked and she does not drink alcohol. The family history was notable for Paget's Disease. She denied any family history of thromboembolism, bleeding, or clotting disorders. Other than Imuran and Fosamax, the patient did not take any other medications.

**Figure 1:**
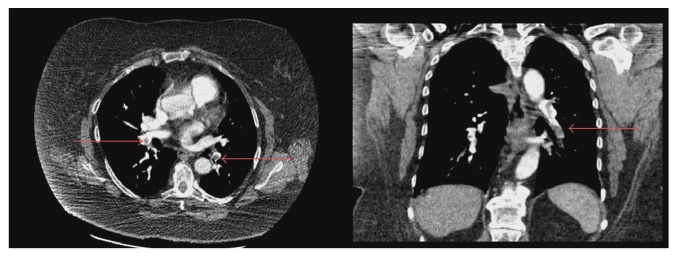
CT angiography showing bilateral pulmonary emboli (red arrows).

**Figure 2:**
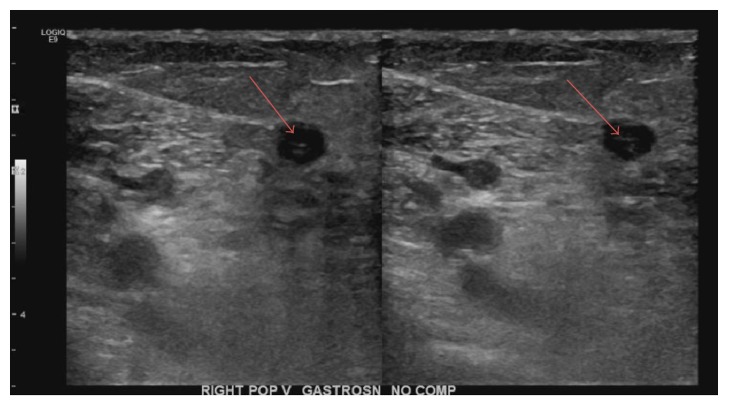
Ultrasound of the right lower extremity showing noncompression of the right popliteal vein as well as a visible thrombus in the lumen (red arrows).

Initial vital signs showed blood pressure of 115/70 torr, heart rate of 125 beats per minute (bpm), respiratory rate of 18 breaths per minute, and temperature of 97.1 F, with oxygen saturation of 96% on room air. Her pain score was 5/10 and she was anxious. On physical exam, the patient appeared mildly anxious with obvious swelling of her right calf. There was moderate right calf tenderness with +1 pitting edema. Extremity pulses were normal bilaterally. On cardiac examination, there were no murmurs. Breath sounds were diminished bilaterally. Diagnostic studies revealed a significantly elevated d-dimer at 13.28 ug/ml, glucose of 131 mg/dL, BUN of 21 mg/dL, troponin of < 0.02 ng/dL, b-type natriuretic peptide (BNP) of 39 pg/mL, and arterial pH of 7.47 and PaO_2_ of 74 on room air (RA). Liver function tests were normal, with an alanine aminotransferase (ALT) of 18 units/L and an aspartate aminotransferase (AST) of 25 units/L, consistent with good control of her autoimmune hepatitis. Her international normalized ratio (INR) was 1.0. Ultrasound revealed occlusive thrombus to right popliteal and right posterior tibial vein (see Figure 1). A Computed Tomography Angiography (CTA) of the chest was positive for bilateral pulmonary emboli (see Figure 2): specifically right upper lobe, right middle lobe, and left lower lobe segmental thrombi. After two liters of intravenous (IV) 0.9% normal saline, her heart rate came down to 91 bpm. Anticoagulation was initiated in the ER with IV heparin. She had a 2D echocardiogram that revealed mild right ventricular dysfunction, mild tricuspid regurgitation, and an elevated pulmonary artery pressure of 25–30 mmHg at rest. The pulmonary/critical team was consulted and given that the patient had normal troponin, BNP, and oxygenation on RA, thrombolytics were withheld. Additionally, the patient's tachycardia improved with IV fluids.

It is uncertain, however, whether the evidence of mild right heart strain was acutely due to the pulmonary emboli or if these findings were chronic and due to the patient's body habitus and sleep apnea. Her general stability and response to IV fluids would suggest the latter.

The patient was hemodynamically stable upon leaving the emergency department for the Intensive Care Unit (ICU). She was transitioned over to a novel anticoagulant rivaroxaban the following day (hospital day number 1) and was discharged home three days later without complications.

## 2. Discussion

In 1984, de Zwaan et al. published a case report in which a 45-year-old male developed a pulmonary embolus after watching television on a couch for consecutive hours. He had a habit of flexing and sitting on one leg as he sat. “Our patient illustrates that venous thrombosis may occur while watching television with 1 leg flexed under the buttocks.” The authors point out that, given the millions of television viewers each day, many of whom watch for consecutive hours, “our patient should be a warning to the avid television watcher to move the legs regularly during the show” [1].

Shirakawa et al., writing for the Japanese Collaborative Cohort (JACC) study group, acknowledged the de Zwaan case report in their study of the relationship between television watching and the risk of mortality from pulmonary embolism. The JACC study is a population-based cohort study involving over 110,000 participants in 45 regions in Japan. Participants were asked about their time spent watching television and were classified into three prespecified categories. The three categories were <2.5 hours/day, 2.5 to 4.9 hours per day, and >5 hours per day. Mortality from pulmonary embolism was obtained from death certificates. Time watching television was positively associated with the risk of mortality from pulmonary embolism. Compared to the <2.5 hours/day group, the hazard ratio for the 2.5–5.9 hours/day group was 1.7 and the risk for the >5 hours/day group was 2.5, establishing a doubling of risk for those in the longest watching category [2].

Shirakawa et al. note that their results are consistent with the Nurses' Health Study data published by Kabrhel et al., in which there was a very strong and linear statistical association between time spent sitting at home and the incidence of pulmonary embolism [3].

In a letter to the editor, Dixon et al. support the JACC study results and suggest that the data is sufficiently strong to warrant “asking patients about their structured exercise frequency and duration as well as their sitting time and inactivity” [4]. Physical inactivity may be a risk factor for cardiovascular disease, “independent of physical activity” [5].

What is the potential mechanism between prolonged time spent sitting and pulmonary embolic disease? It has been argued that the mechanisms discussed in the literature concerning the risk of long distance travel (air, automobile) may apply to prolonged time spent sitting. These involve venous stasis predominantly—with some evidence that prolonged sitting may increase hypercoagulability [6].

What can be done? Chevance noted that “regularly interrupting the sedentary behavior has favorable effects on health, regardless of the time spent sitting” [7].

Interruption of time spent sitting may have other benefits. Time spent sitting has been shown to be related to total mortality, especially due to cardiovascular causes [8], and has been linked to a higher overall risk of cancer in women [9].

The advent of streaming technology has made what is colloquially referred to as “binge watching” through TV and other digital devices easy and common. It has always been the goal of technology to decrease human analog input, and as a result, the decline in the physical activity in our delay living may have translated into an even more sedentary lifestyle. In the span of several decades, we went from having to get up to change channels to having thousands of movies and multiple seasons of TV series in the luxury and comfort of our tablets and smart phones.

Our patient's vascular event clearly illustrates the risk of thromboembolism from prolonged TV consumption, especially if a preexisting risk factor such as obesity already exists. The medical community and the public may well be aware that traveling for a long period of time is a risk. Now, we should add a reminder of the same risk when watching television.

## 3. Conclusions

Death from pulmonary embolus while watching TV is a rare phenomenon. While growing evidence is available regarding the association between sedentary lifestyle and obesity, diabetes, and cancer, thromboembolic events may be added to the list. More studies as well as education of the public are needed to spread awareness regarding the possible danger of too much TV and PE/DVT.
